# Improving Short Term Instability for Quantitative Analyses with Portable Electronic Noses

**DOI:** 10.3390/s140610514

**Published:** 2014-06-13

**Authors:** Miguel Macías Macías, J. Enrique Agudo, Antonio García Manso, Carlos Javier García Orellana, Horacio Manuel González Velasco, Ramón Gallardo Caballero

**Affiliations:** 1 University Center of Merida, University of Extremadura, Sta. Teresa de Jornet, 38, Mérida 06800, Spain; E-Mail: jeagudo@unex.es; 2 Polytechnic School, University of Extremadura, Cáceres 10003, Spain; E-Mails: antonio@capi.unex.es (A.G.M.); horacio@capi.unex.es (H.M.G.V.); ramon@capi.unex.es (R.G.C.); 3 Faculty of Science, University of Extremadura, Avda, Elvas s/n, Badajoz 06006, Spain; E-Mail: carlos@capi.unex.es

**Keywords:** electronic nose, quantitative analysis, gas sensor

## Abstract

One of the main problems when working with electronic noses is the lack of reproducibility or repeatability of the sensor response, so that, if this problem is not properly considered, electronic noses can be useless, especially for quantitative analyses. On the other hand, irreproducibility is increased with portable and low cost electronic noses where laboratory equipment like gas zero generators cannot be used. In this work, we study the reproducibility of two portable electronic noses, the PEN3 (commercial) and CAPINose (a proprietary design) by using synthetic wine samples. We show that in both cases short term instability associated to the sensors' response to the same sample and under the same conditions represents a major problem and we propose an internal normalization technique that, in both cases, reduces the variability of the sensors' response. Finally, we show that the normalization proposed seems to be more effective in the CAPINose case, reducing, for example, the variability associated to the TGS2602 sensor from 12.19% to 2.2%.

## 1. Introduction

During the last four decades a lot of research aimed at developing olfactory electronic systems or electronic noses has been conducted. During this time, automatic odour detection systems have been applied in many industrial applications, agriculture [1], indoor air quality [2], environmental monitoring [3], quality control of food products [4,5], medical diagnosis [6], as well as many others. On the other hand, some developments have been directed towards the construction of low cost and compact electronic noses [7,8] which have led to a number of commercial e-nose products [9].

An electronic nose is a device that can smell in a similar way to a human nose and is able to perform qualitative and/or quantitative analysis of a mixture of gases, vapours and odours. It can distinguish and recognize scents by using different sensors. A device of this type has at least four parts with different functions: the first ensures the adequacy of the gas mixture and sampling, the gas sensor array performs the detection, the control electronics part is dedicated to the management of the sensor array and adequacy of the signal, and, finally, a computer with suitable pattern classification algorithms, extracts the characteristic features of each scent and presents the results on the user interface.

The usual process of identifying odours comprises three states during odour sampling: the first cleans the measuring chamber and sensors with “clean air or zero gas” to sweep the molecules of previous measurements, the second is the sampling, where odour samples are received in the chamber exposing the sensors to it, and finally a second cleaning of the chamber in preparation for the next sample takes place. Thermal stabilization of the sample and the chamber during all these stages is usually required. Similarly, as the humidity affects both the measurement and the sample itself results should only be taken and compared under standard conditions of humidity and temperature.

When a volatile compound comes into contact with the surface of the sensor array, a set of physical changes modifies the properties of the material from which each sensor is composed. This perturbation can be measured, digitalized and used as a feature for the specific compound [10].

One of the major problems when working with electronic noses lies in the instability (irreproducibility or variability) of the sensor responses. This lack of stability over time limits the widespread adoption of artificial olfaction systems in real industrial setups [11]. This problem of chemical sensor stability over time is known as sensor drift, which consists of small and non-deterministic temporal variations of the sensor response when it is exposed to the same sample under the same conditions [12].

Long term instability (instability when we consider measurements taken on a period of time of several months or years) can be due to sensor aging [13] or thermomechanical degradation [14]. Short term instability (instability when we consider measurements taken over a period of time of several days or weeks) can be influenced by a variety of sources, including environmental factors [15] or deficiencies in the hardware of the electronic nose system.

Short term instability can be partially corrected by using laboratory equipment like gas zero generators or flowmeters but some of these tools are difficult or impossible to include in the design of portable electronic noses. This instability can also be improved by internal normalization, for example, expressing each sensor response as a percentage of the sensor with the maximum intensity [16]. Although this data treatment partially corrects irreproducibility, quantitative information is lost after normalization making these techniques useless for quantitative analysis, namely, this normalization is only appropriate for those problems in which the shape of the profile signal or fingerprint, and not the intensity of the signals, contains the relevant information.

Long term instability can be improved with several drift compensation methods [10,17] like baseline manipulation [18], periodic calibration [19,20] or attuning methods [15]. Nevertheless, the study of sensor drift is still a challenging task for the chemical sensor community [11].

In this paper, the short term instability of two portable electronic noses, the PEN3 electronic nose by Airsense and the CAPINose electronic nose designed by our research group is analysed. To achieve this objective, we have used synthetic wine matrices (aqueous ethanol solutions with a 10% V/V ethanol content), proposing later a normalization technique that reduces the short term instability of the sensor response of the electronic noses without losing the quantitative information of the samples. Finally we put forward hardware arrangements that can help improve the short term instability of the electronic noses.

The general characteristics of the electronic noses used are shown in Section 2. We test the short term instability of the sensor responses in both electronic noses in Section 3. The normalization technique is discussed in Section 4 and finally, in Sections 5 and 6 the results and conclusions of the current work are presented.

## 2. Hardware

### 2.1. PEN3 Electronic Nose

One of the electronic noses used in this work is a portable electronic nose (PEN3), made by Win Muster Airsense (WMA) Analytics Inc. (Schwerin, Germany). This system has been used for different purposes, for example, to characterize peach cultivars and to monitor their ripening stage [21], to analyze volatile emissions from wastewater [22] and to monitor the storage shelf life of tomatoes [23].

The portable electronic nose (PEN) consists of a sampling apparatus, a detector unit containing the array of sensors, and pattern recognition software (Win Muster v.1.6) for data recording and elaboration. The sensor array is composed of 10 metal oxide semiconductor (MOS)-type chemical sensors and the sensor response is expressed as resistivity (Ohm) and relies on changes in conductivity induced by the adsorption of molecules in the gas phase, and on subsequent surface reactions.

Measurements are taken by using the dynamic headspace technique [24], sampling in which the headspace of the vials is continuously swept into the detector by a clean purge flow for analysis. This way, the gaseous analyte concentration immediately above the liquid phase is kept as low as possible to increase the evaporation rate. This evaporation rate depends on the surface area, the analyte surface concentration, the analyte volatility and the sample temperature.

**Figure 1.**
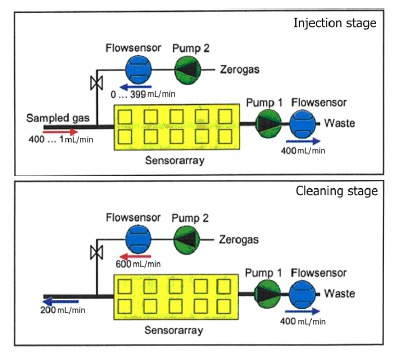
Schematic diagrams of the gas flow of PEN3 during the electronic nose measurements.

The measurement phase of the electronic nose is divided into two stages, injection and cleaning. The volatiles of the sample are transported to the sensor chamber to be analyzed by the array of sensors in the injection phase, and all traces of volatiles must be removed of the electronic nose to avoid interference with the next measurement in the cleaning phase. We can observe the gas flow in each stage in Figure 1. Pump 1 sucks the sample gas compounds through the sensor array and Pump 2 transfers filtered reference air into the sensor array in the injection phase. This arrangement allows dilution and avoids saturation of the sensors due to, among other reasons, high concentrations of ethanol. In the cleaning phase, the cleaning air flow of Pump 2 is used to rinse the system. Due to the higher flow rate of Pump 2, the original gas flow direction at the inlet is inverted.

There are a lot of parameters that have to be adjusted in order to find a suitable response of the electronic nose. The most important ones are the injection time, the flush or cleaning time and the injection flows of the sampled gas, the zero gas and the waste gas. After numerous tests, measurements showed in this paper were taken with the values of the adjustable parameters of the PEN3 electronic nose as follows:

■ Injection sampled gas: 20 mL/min.
■ Zero gas flow: 380 mL/min.
■ Waste flow: 400 mL/min.
■ Injection time: 3 s.
■ Cleaning time: 300 s.
■ Measuring interval: 0.2 s.

### 2.2. CAPINOSE Electronic Nose

**Figure 2.**
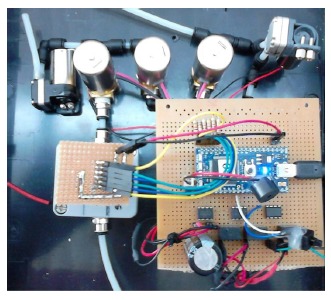
CAPINose electronic nose.

The CAPINose electronic nose is a portable electronic nose that was presented in [8]. As we can see in Figure 2, the electronic nose hardware is composed of two small pumps, three electro-valves and a chamber containing five sensors which are connected and controlled by an MBED microcontroller [25].

**Figure 3.**
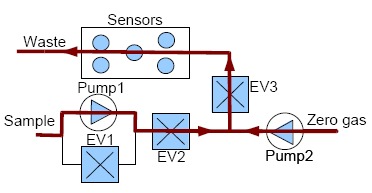
Gases flow at the injection stage.

**Figure 4.**
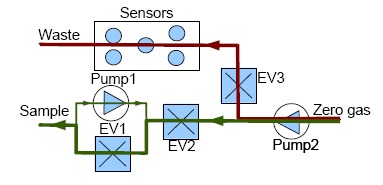
Gases flow at the cleaning1 (red) and cleaning2 (green) stages.

Measurements with our e-nose are also taken by using the dynamic headspace technique and each measurement with our electronic nose is divided into two stages, *injection and cleaning*. The voltage applied to the pumps controls the amount of gas flow and the dilution at the injection stage while the electro valves control the path of the gases. In addition, to ensure the cleaning of the entire system (the sensor chamber and the other components of the electronic nose), the cleaning stage is divided into two sub stages: *cleaning1* and *cleaning2*. The flow of gases in the injection and cleaning stages can be observed in Figures 3 and 4, respectively.

The electrovalve marked as EV2 is not only used for controlling the path of the gases, but also to control the amount of the sample gas entering the sensor chamber more accurately.

Five TGS Figaro gas sensors, TGS26XX (with XX = 00, 02, 20, 20, 11) obtained from Figaro Engineering, Inc. (Osaka, Japan) have been used in CAPINose. These sensors show a certain degree of affinity towards a specific gas but are sensitive towards a wide spectrum of gas types with overlapping sensitivities. These sensors have been mounted in a stainless steel sensor chamber. A 1 KΩ load resistor is connected in series with each sensor and a common power supply of 5 V is used for both the heater voltage of the sensors and the voltage applied to the voltage divider formed by the sensors and the load resistances. Finally, the response of the sensor is the voltage measured at the output of the voltage divider. We have used two TGS2620 units to verify that the two sensors have similar irreproducibility values.

As in the case of the PEN3, fresh air is used as a carrier gas and measurements start when the sensor resistances in the presence of the carrier gas are stabilized. Measurements were taken with the following values of the adjustable parameters of CAPINose:

- Pump1 voltage: 3 V
- Pump2 voltage: 5 V
- Cleaning1 stage time: 200 s
- Cleaning2 stage time: 100 s
- Injection stage time: 3 s
- Measurement interval: 1 s

## 3. The Reproducibility Test

### 3.1. Variability Measure

To test the short time instability of the two electronic noses we used synthetic wine samples, made up of aqueous ethanol solutions with a 10% V/V ethanol content. Measurements were taken on five different days with a time gap of seven days between them. The measurement process was repeated several times each day.

**Figure 5.**
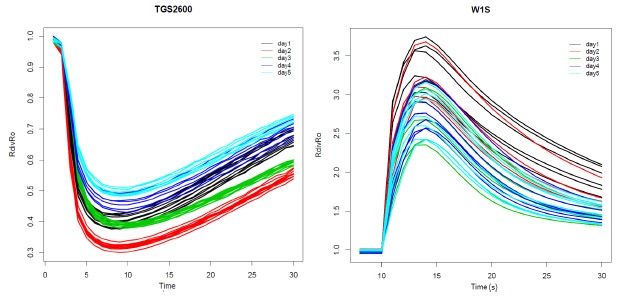
*R*/*R_o_* curves for the TGS2600 sensors in the CAPINose and W1S ones in the PEN3.

We can observe examples of the sensor response curves for the TGS2600 installed in CAPINose and W1S installed in the PEN3 in Figure 5. Each sensor response curve represents the values of the resistance of the sensor *R* over time. This resistance has been divided by the baseline (initial value of the resistance *R_o_*) [18]. Different colors are used for the *R*/*R_o_* curves taken on different days. We can observe the irreproducibility associated to the measurements of the same sample and under the same conditions in this figure. We can also observe that irreproducibility is lower for measurements taken on the same day (we call it the intraday variability) and it increases when we consider all measurements (we call this the interday variability).

To estimate the variability associated to a set of measures as shown in Figure 5, we must first choose a feature representative of each sensor curve and then we must calculate it for each sensor curve. Finally, we must define a figure of merit that quantifies the variability of the feature values. Considering that the features selected can take values at different scales, a normalized measure of dispersion that takes into account the magnitude of the feature should be used. In that sense, to quantify the variability of such features, the coefficient of variation (CV) will be used [26], that is defined as the ratio of the standard deviation to the mean, showing the extent of variability in relation to the population mean. On the other hand, we have used boxplots to visually compare the variability associated to a set of distributions. Boxplots are a way of graphically depicting groups of numerical data through their quartiles. The spacing between the different parts of the box helps to indicate the degree of dispersion and skewness in the data and identify outliers [27].

### 3.2. Feature Extraction

Each sensor response curve of the Figure 5 is composed of N points corresponding to the measurements of the sensors in the injection and cleaning phases. A lot of features have been proposed for the characterization of these sensor response curves: maximum, minimum, slope, average, among others. In [28], we used principal component analysis (PCA) [29] to reduce the dimensionality of the N-dimensional vector that initially characterizes the sensor response. We showed in that work that only the first PC accumulated 96.5% of the initial variance, so we only used this component to characterize each response curve. Due to when all measurements are positively correlated, the first principal component is often some kind of average of the measurements, for simplicity, the average feature instead of the projection of the sensor response curve onto the first principal component is used in this work. Therefore, we have chosen three features to characterize the *R*/*R_o_* sensors curves:

- AVR: Average of the *R*/*R_o_* values over the time.
- EXT: minimum or maximum of the *R*/*R_o_* values, depending on whether the sensor response curves decrease or increase on the presence of the sample gas.
- RG: defined as the absolute value of the difference between the maximum and the minimum of the *R*/*R_o_* values.

**Figure 6.**
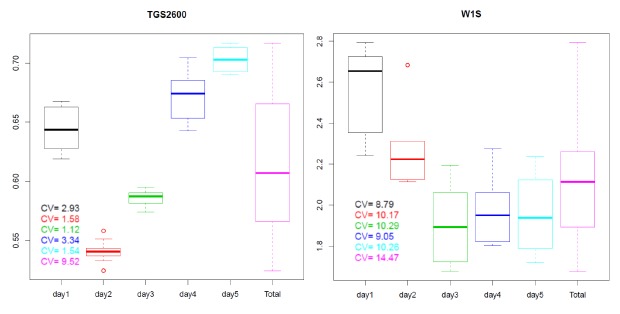
Boxplots of the distributions of the AVR feature for the *R*/*R_o_* curves of Figure 5.

In Figure 6 the boxplot of the distributions of the AVR feature calculated for the *R*/*R_o_* curves shown in Figure 5 can be observed. The boxplots for the measures taken on different days are represented in different colors. The values of the variation coefficient for each distribution are also shown.

As we can see in Figure 6, the variability associated to the AVR feature is low for measures taken on the same day (intraday variability) with values ranging from 1.12% (day3 in green) to 3.34% (day4 in blue) for the TGS2600 of the CAPINose. However, the intraday variability takes values from 8.79% (day1 in black) to 10.29% (day3 in green) for the W1S sensor of the PEN3. We can also observe that variability increases greatly when measures taken in different days are considered together (interday variability) taking values of 9.52% for CAPINose and 14.47% for PEN3.

Although the boxplots of Figure 6 correspond to one feature (AVR) and one sensor for each electronic nose, as we will show in Section 5, these results could be extrapolatable if we had considered another feature and sensor. This variability or short term instability can render useless the electronic noses to distinguish samples that only vary in the concentration of one of its components.

## 4. Normalization

**Figure 7.**
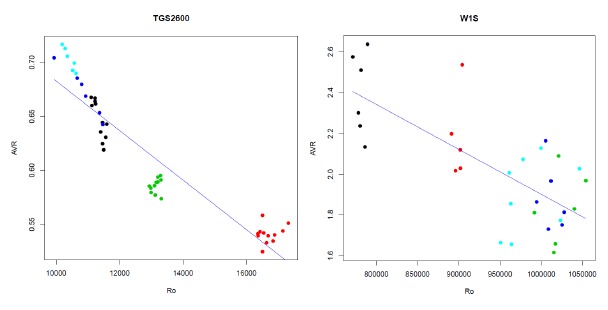
Dependence of AVR, calculated over the sensor curves of the Figure 5, on the values of *R_o_*.

A normalization scheme that, especially for some sensors and features, greatly reduces the variability is proposed in this section. We can observe in Figure 7 what usually happens when we represent the values of the features (AVR for TGS2600 in the CAPINose and AVR for the W1S in PEN3) *versus* the values of the initial resistance of the sensor *R_o_*. As we can observe, a very clear linear dependence exists. We have used this dependence to normalize and reduce the variability.

If we use simple linear regression [30] to express the dependence of the feature *F* as a linear function of *R_o_*, we have to estimate the coefficients *a* and *b* of equation one obtaining the blue lines of the diagrams in Figure 7. Then we can use expression two to obtain a new feature *F_c_* corrected from the dependence of *R_o_*:

(1) $$F=a+bR_{o}$$

(2) $$F_{c}=F-b(R_{o}-{\bar{R}}_{o})$$

**Figure 8.**
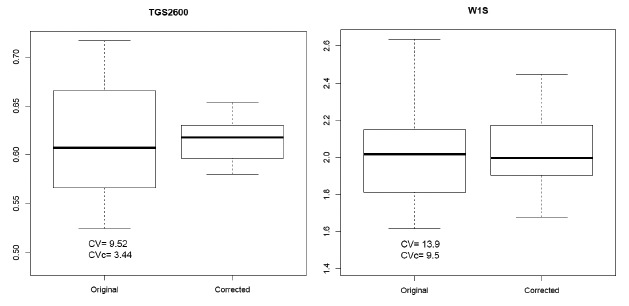
Boxplot of the distributions of the values of AVR of Figure 5 (original) and its normalized values (corrected).

We can observe in Figure 8 the boxplots of the distributions of the AVR (original) and AVR_c_ (corrected by normalization) features represented in Figure 7 and the values of the coefficient of variation of the AVR and AVR_c_ features. In these cases, the normalization has reduced the value of the *CV* from 9.52% to 3.44% in the CAPINose case and from 13.9% to 9.5% in the PEN3 case.

## 5. Results

The values of the coefficient of the variation CV for the features EXT, AVR and RG are calculated for all the sensors in the PEN3 and CAPINose electronic noses in this section. In the PEN3 case, only the six sensors (W1S, W5S, W3C, W5C, W4S and W2S) more sensible to ethanol are considered. The value of the coefficient of variation *CV* of each feature is also compared with its normalized value *CV_c_*.

Besides, the mentioned values are calculated over the *R* (without baseline manipulation) and over the *R/R_o_* (with baseline manipulation) sensor response curves. The values of *CV* and *CV_c_* for the features AVR, EXT and RG calculated over the *R*/*R_o_* and *R* curves of the CAPINose sensors are showed in Table 1. In Table 2, the same values are calculated for the sensors of PEN3. The feature with the least value of *CV_c_* for each sensor is shadowed.

## 6. Conclusions

We propose in this paper a normalization scheme based on the dependence of the calculated features with the values of the initial resistance of the sensor *R_o_*. As we can observe in Tables 1 and 2, this normalization partially reduces the instability associated to the sensors' response. For example, the *CV* of the *RG* feature calculated over the *R* curves of the TGS2602 sensor of the CAPINose is 12.19% and it is reduced to 2.2% by using the proposed normalization. The normalization also reduces the variability associated to the PEN3 sensors but in a lesser extent than in the CAPINose case. In our opinion CAPINOSE intraday variability is smaller because the electrovalve (EV2) helps to control more accurately the amount of volatiles entering the sensor chamber and this causes the initial data to be easier to normalize.

According to the values of the *CV_c_* showed in Tables 1 and 2, we can also say that the features calculated over the *R* curves have less variability than the ones calculated over the *R*/*R_o_* curves. On the other hand, *CV* and *CV_c_* values associated to the sensors of the PEN3 are greater than those of the CAPINose sensors. This may be due to the fact that the proposed normalization overall corrects interday variability more than intraday variability and as we showed in Figure 6, the intraday variability for the PEN3 is greater than for the CAPINose.

## Figures and Tables

**Table 1.** *CV*/*CV_c_* values for the sensors of CAPINose.

**CAPINose (*CV*/*CV_c_*)**	***R*/*R_o_***			***R***		
		
**Sensor**	**AVR**	**EXT**	**RG**	**AVR**	**EXT**	**RG**
**TGS2600**	9.5/3.44	14.88/6.2	9.73/4.2	9.26/2.4	6.2/4.97	27.71/3.4
**TGS2602**	9.9/6.1	14.5/9.5	3.47/2.3	5.99/5.6	9.08/9.01	12.19/2.2
**TGS2620-1**	9/3.2	14.74/6.6	9.6/4.4	8.76/2.4	6.39/5.59	26.23/3.5
**TGS2620-2**	9.1/3.8	15.23/7.2	9.21/4.4	8.4/2.8	6.21/5.83	25.43/3.5
**TGS2611**	1.1/1.1	2.31/2.3	6.38/6.2	3.82/1.1	4.99/2.29	6.89/6.3

**Table 2.** *CV/CV_c_* values for the sensors of PEN3.

**PEN3 (*CV*/*CV_c_*)**	***R*/*R_o_***			***R***		
		
**Sensor**	**AVR**	**EXT**	**RG**	**AVR**	**EXT**	**RG**
**W1S (S1)**	4.9/4	12.3/10.1	10.2/8.3	17.8/3.7	22.7/9.2	12.7/7.7
**W5S (S2)**	13.9/9.5	12.8/9.6	19.1/14.3	9.5/9.4	9.9/9.6	14.5/14.4
**W3C (S3)**	5.9/5.8	13.9/13.4	11.6/11.1	12/5.6	18/12.7	13/10.7
**W5C (S5)**	5.6/5.1	13.5/11.3	7.1/6	5.4/5.1	11.8/11.3	10.2/5.9
**W4S (S6)**	7.5/7	9.9/8.7	11.5/10.2	14.2/7.1	13.8/8.8	14.4/10.3
**W2S (S8)**	9.7/8.8	17.3/14.3	21.2/17.7	10.3/8.8	14.2/14.2	17.7/17.6
